# Clinical presentation, treatment, and outcome of children with primary intestinal lymphangiectasia: A national retrospective study

**DOI:** 10.1002/jpn3.70266

**Published:** 2025-11-23

**Authors:** Noémie Goret, Cécile Lambe, Emmanuelle Ecochard‐Dugelay, Alexandre Fabre, Alain Dabadie, Aurélie Comte, Julie Rebeuh, Pierre Poinsot, Emilie Chaillou, Haude Clouzeau, Aurélie Sabard, Béatrice Dubern, Stéphanie Willot

**Affiliations:** ^1^ Service de Médecine Pédiatrique, CHU de Tours Tours France; ^2^ Service de Gastroentérologie, Hépatologie et Nutrition Pédiatrique, Hôpital Necker – Enfants Malades Université Paris Cité Paris France; ^3^ Service de Gastroentérologie, Hépatologie et Nutrition Pédiatrique, APHP Robert Debré Paris France; ^4^ APHM, Hôpital de la Timone Enfant, Service de Pédiatrie Multidisciplinaire Marseille France; ^5^ Pédiatrie, CHU Rennes Rennes France; ^6^ Service de Pédiatrie, CHU Besançon Besançon France; ^7^ Service de Pédiatrie Hôpitaux Universitaires de Strasbourg Strasbourg France; ^8^ Service de Gastroentérologie, Hépatologie et Nutrition Pédiatrique, Hospices Civil de Lyon, Hôpital Femme Mère Enfant Lyon France; ^9^ Département de Pédiatrie médicale, CHU Angers Angers France; ^10^ Service de Gastropédiatrie, CHU de Bordeaux, Hôpital Pellegrin‐Enfants Bordeaux France; ^11^ Service de Nutrition et Gastroentérologie Pédiatrique, APHP Trousseau Sorbonne Université Paris France

**Keywords:** dilated intestinal lymphatics, pediatric Waldmann's disease, protein‐losing enteropathy

## Abstract

**Objectives:**

Primary intestinal lymphangiectasia (PIL) is a very rare disease responsible for protein‐losing enteropathy. There is little published data about treatments efficacy and outcomes. Our main objective was to describe the clinical profile, response to therapy, and outcomes of children with PIL.

**Methods:**

We conducted a national retrospective study including children with PIL followed in French university hospitals between 2010 and 2022. Response to treatment was defined as clinical remission and no need for albumin infusion. Response was considered complete if albuminemia was normal (>35 g/l) during follow‐up and partial if less than 35 g/l.

**Results:**

Thirty‐four children (22 males) were included; median age at diagnosis was 7 (3–29,5) months. The median follow‐up in our cohort was 4.5 years. Edema (79%), chronic diarrhea (50%), and ascites (35%) were the main symptoms. Thirty‐one patients received a low long‐chain triglycerides dietary therapy and 25 (81%) responded: 15 had a partial response and 10 a complete response. Four patients (12%) required a second‐line drug treatment. The presence of lymphedema or an identified genetic variant were associated with a partial response to diet (*p* < 0.05). A normal diet could be reintroduced in 14 patients (45%) without relapse during the follow‐up. Among them, nine children (26%) were considered cured with a complete and prolonged remission under a normal diet.

**Conclusions:**

Most children with PIL responded to diet therapy and about a quarter of the cohort had a good prognosis with complete remission even after discontinuation of the diet. Presence of lymphedema or a genetic variant was associated with a chronic condition.

## Abstract (highlights)

**What is Known:**

- Dietary management is usually the first treatment for primary intestinal lymphangiectasia (PIL) and the majority of children respond to it.
- The utilization of second‐line treatments remains a subject of controversy in the literature.

**What is New:**

- We describe two distinct evolution profiles for children who have a clinical response to the diet. The majority exhibited a partial response with persistent asymptomatic hypoalbuminemia, while some demonstrated a complete response with rapid and stable normalization of albumin levels.
- Use of second‐line treatments is infrequent in our cohort (12% of patients).

## Graphical abstract

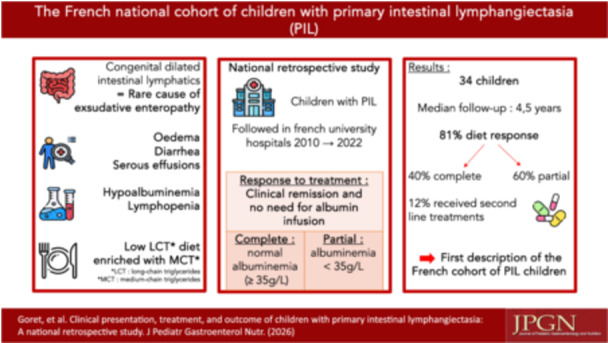

## 1 INTRODUCTION

Primary intestinal lymphangiectasia (PIL) is a rare congenital disease characterized by idiopathic dilated intestinal lymphatics responsible for protein‐losing enteropathy with lymphopenia, because of chyle leaking in the intestinal lumen. It is associated with clinical symptoms such as edema, chronic diarrhea, serous effusions, and biological features: hypoalbuminemia, hypogammaglobulinemia, and lymphopenia.^1^, ^2^, ^3^, ^4^ Gastrointestinal protein loss is assessed by measuring increase of alpha1 antitrypsin clearance. The diagnosis of PIL is usually made in the first years of life and requires esophagogastroduodenoscopy, ileocolonoscopy or videocapsule endoscopy, looking for suggestive findings: small whitish patches, or diffuse prominent white villi in the duodenum and/or ileum, with dilated lymphatic channels on biopsies.

The first‐line treatment is based on a strict dietary therapy composed of a low long‐chain triglycerides (LCT) diet enriched with medium‐chain triglycerides (MCTs) and high protein intakes.^5^, ^6^ The low LCT diet helps to reduce lymphatic pressure in the splanchnic region. This decrease in pressure minimizes the dilation of lymphatic vessels and chyle leakage in the intestinal lumen. The inclusion of MCTs in the diet ensures a lipid intake that bypasses the lymphatic circulation, as MCTs are directly absorbed through the hepatic portal vein system. Regular monitoring of fat‐soluble vitamin levels and essential fatty acids (via chromatography of essential fatty acids) is crucial during this diet to detect potential deficiencies associated with a fat‐free diet.

In case of nonresponse with persistent symptoms and severe hypoalbuminemia requiring iterative infusions, second‐line drug treatments such as octreotide or sirolimus can be discussed.^7^, ^8^ However, these remain controversial. The most recent studies have focused on providing individual treatment recommendations based on the location and extent of the disease.^9^, ^10^, ^11^, ^12^ Since Waldmann reported the first case of intestinal lymphangiectasia in 1961, fewer than 200 cases have been reported worldwide. Due to the rarity of this pathology, prospective studies are not possible and articles in the literature are essentially case reports focusing on the diagnosis, with poor data on the follow‐up and outcome of these patients.^13^, ^14^, ^15^, ^16^, ^17^, ^18^ We therefore decided to conduct a national multicentric retrospective study, of which the main objective was to describe the French national cohort of children with PIL, with a focus on clinical presentation, response to treatments, and outcomes. The secondary objective was to define, at the time of diagnosis, the clinical and biological characteristics that could be associated with a complete response to dietary therapy.

## 2 METHODS

### 2.1 Ethics statement

This retrospective study received approval from the Ethics Committee of the Groupe Francophone d'Hépatologie‐Gastroentérologie et Nutrition Pédiatrique (GFHGNP) under Review 2022‐43. Information letters tailored for children and their families were provided to ensure their non‐opposition to the research.

### 2.2 Study design

All pediatric gastroenterologists from the French medical centers specialized in the management of rare digestive diseases were invited to participate in this study. We included children (less than 18 years old) diagnosed with PIL and followed in those centers from January 2010 to January 2022. All secondary causes of intestinal lymphangiectasia, including infectious, inflammatory, and cardiac conditions were excluded. We ensured accuracy by relying on the expertise of investigators at each center, followed by an independent review of the medical records by the coordinating team. We also excluded conditions involving widespread lymphatic involvement without protein‐losing enteropathy. After exclusion of secondary causes, the diagnosis was most often confirmed by histological or endoscopic results. In cases of noncontributory endoscopy, diagnostic confirmation required the presence of at least one of the two following criteria: genetically confirmed Hennekam syndrome (typically linked to PIL) associated with protein‐losing enteropathy (increased clearance of alpha1‐antitrypsin >24 mL/day and lymphopenia) or protein‐losing enteropathy with good response to dietary therapy. For efficient data management in this multicenter study, each investigator was invited to fill‐in details of the patients' charts on a certified secure data warehouse. All diagnosis data, including demographic details, symptoms, anthropometry (weight, height), laboratory results, imaging, endoscopy and pathology, were collected. Age‐appropriate standard thresholds were applied to define hypoalbuminemia, lymphopenia, and hypogammaglobulinemia. Details regarding dietary compliance, symptoms progression, laboratory results (albumin, lymphocytes count, and Immunoglobulin G levels), growth parameters (using national french growth charts AFPA‐CRESS/INSERM‐CGM), and complications were recorded at 2 months, 6 months, and 1 year postdiagnosis, and subsequently on an annual basis until January 2022. In our study, response to treatment (diet and/or drugs) was defined as clinical remission corresponding yo the prolonged resolution of symptoms including edema, diarrhea, asthenia, abdominal pain, serous effusions, inappropriate weight loss or gain, without the need for albumin infusion. The indication for albumin infusion was at the discretion of the practitioner. Among patients who responded, complete response was defined as the sustained normalization of albumin concentration (albuminemia ≥ 35 g/L), while a partial response was defined as a persistent hypoalbuminemia during follow‐up (albuminemia < 35 g/L). Quantitative variables were presented as median values and interquartile range, while categorical variables were expressed as numbers and percentages. Categorical variables were compared using the chi‐squared test (with Fisher exact test wherever applicable), and continuous variables were assessed using the Mann–Whitney *U* test. A significance level of *p* < 0.05 was adopted.

## 3 RESULTS

**Figure 1:**
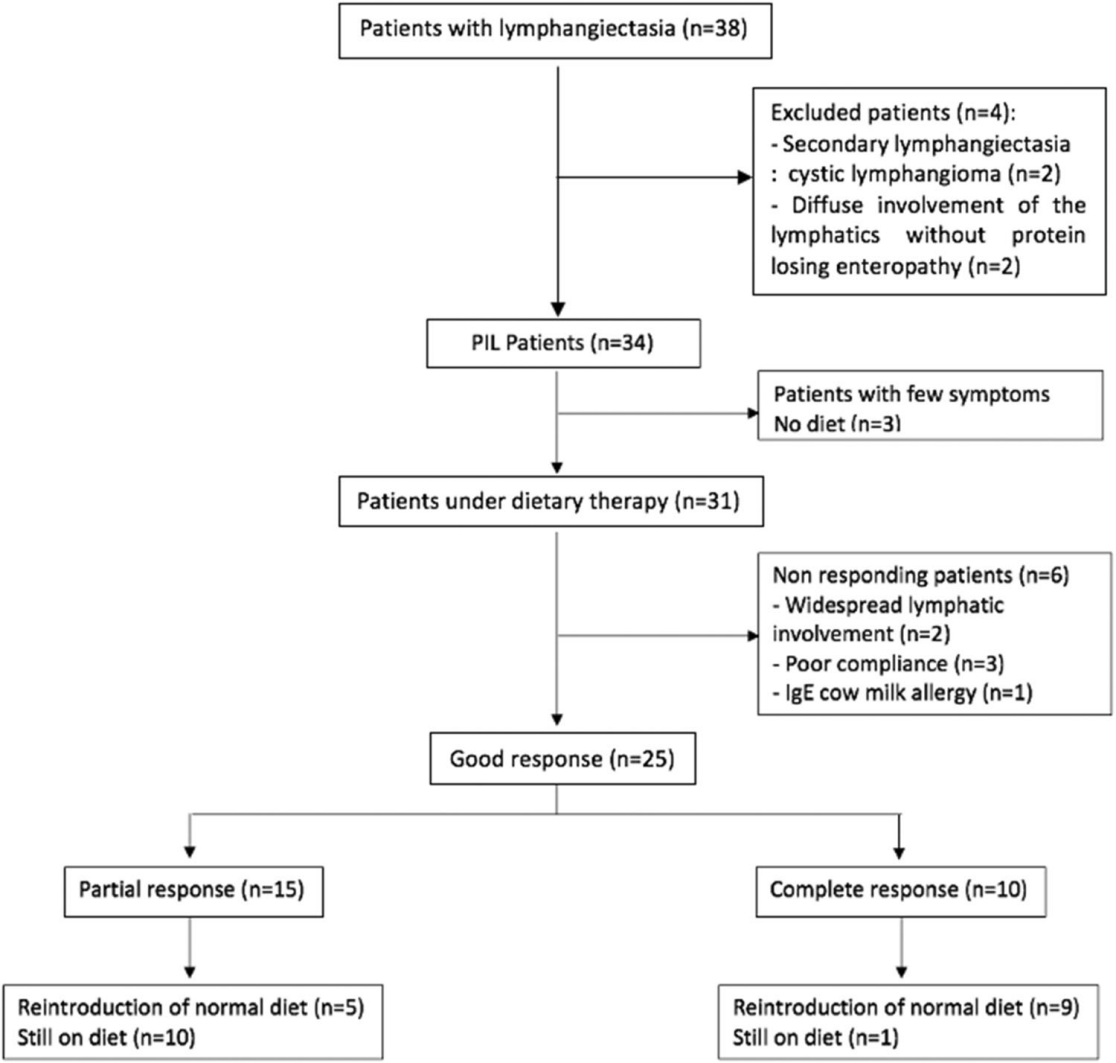
Flow chart of patients with PIL and their outcome during the dietary treatment. PIL, primary intestinal lymphangiectasia.

We received answers from 33 (92%) out of the 36 French medical centers officially part of the national network for rare digestive diseases. Eleven centers followed patients with lymphangiectasia: University Hospitals of Paris (Necker [*n* = 10], Robert Debré [*n* = 6], Trousseau [*n* = 3]), Tours (*n* = 5), Marseille (*n* = 4), Besançon (*n* = 2), Lyon (*n* = 2), Rennes (*n* = 2), Strasbourg (*n* = 2), Angers (*n* = 1) and Bordeaux (*n* = 1) for a total of 38 children. Four children were excluded (Figure 1) because they did not fit the inclusion criteria. In all, 34 children with PIL were included. The main characteristics of patients at diagnosis are described in Table 1 and the diagnostic confirmation method is described in Table 2.

**Table 1:** Patient's characteristics at diagnosis.

		(*n* = 34)
Sex:	Male	22 (65%)
	Female	12 (35%)
Median age:		7 months (3–29.5)
	− **<24 months**	**23 (68%)**
	− >24 months	11 (32%)
Diagnosis delay:		1 month (0.8–12)
Syndromic forms		7 (21%)
	− 2 Unlabeled polymalformative syndromes	
	− 3 Hennekam syndromes	
	− 2 Heterozygous CCBE1 variant without lymphedema	
Growth (standard deviation)		
	− Height/age	+0.2 SD (−2.2; +1.4)
	− Weight/expected weight for height	−0.23 SD (−0,8; +1.7)
Symptoms:		
	− **Edema**	**27 (79%)**
	− **Chronic diarrhea**	**17 (50%)**
	− **Asthenia**	**12 (35%)**
	− **Ascite**	12 (35%)
	− Lymphedema	10 (29%)
	− Anasarca	2 (6%)
Biology:		
	− **Hypoalbuminemia**	**34 (100%)**
	− Albuminemia (35–54 g/L)	19 (16–24)
	− Lymphopenia (2500–5400/µL)	19/28^a^ (68%)
	− **Decreased IgG**	**29/29** a **(100%)**
	− IgG (6–15 g/l)	1.3 (0.7–2.2)
	− Decreased IgM (0.5–1.6 g/L)	19/27^a^ (70%)
	− Decreased IgA (0.4–2.6 g/L)	11/27^a^ (41%)
	− **Increased AAT clearance (>24 mL/d)**	**21/21** a **(100%)**

*Note*: All values are expressed as number (%) or median (Interquartile range). The bold values represent the most frequent characteristics at diagnosis in these patients.

Abbreviations: AAT, alpha‐1‐antitrypsin; CCBE1, Collagen‐ and calcium‐binding EGF domain‐containing protein 1; IgA, immunoglobulin A; IgG, immunoglobulin G; IgM, immunoglobulin M.

^a^ Detailed count report in case of missing data.

**Table 2:** Diagnostic confirmation of the 34 patients with LIP.

Confirmed by endoscopic and/or histologic results	*n* = 27/34 (79%)
Suggestive esophagogastroduodenoscopy and histologic results	*n* = 18 (53%)
Confirmed on histologic result only	*n* = 4 (12%)
Colonoscopy (terminal ileal lymphangiectasia on biopsies)	*n* = 2 (6%)
Confirmed on capsule endoscopy only	*n* = 3 (8%)

*Note*: Genetically confirmed Hennekam syndrome (typically linked to primary intestinal lymphangiectasia) associated with protein‐losing enteropathy evidenced by elevated alpha‐1‐antitrypsin clearancen = 3/34 (9%), *n* = 3/34 (9%). Protein‐losing enteropathy evidenced by elevated alpha‐1‐antitrypsin clearanceassociated with good response to dietary management *n* = 4/34 (12%).

Upon diagnosis, severe hypoalbuminemia necessitated at least one albumin infusion in 78% (*n* = 26) of patients, and several times for most of them with a median of 5 (2–11) infusions per patient. Seven patients (21%) received parenteral nutrition for a median duration of 4 weeks (1.5–14) due to profuse diarrhea accompanied by malnutrition. An infection was documented at the time of diagnosis in 35% of cases (*n* = 12). Among these, three children experienced severe infections, including enterococcal faecalis bacteremia, pneumococcal meningitis, and disseminated cryptococcosis with pulmonary involvement and septicemia. All had a favorable outcome without any long‐term complications.

**Figure 2:**
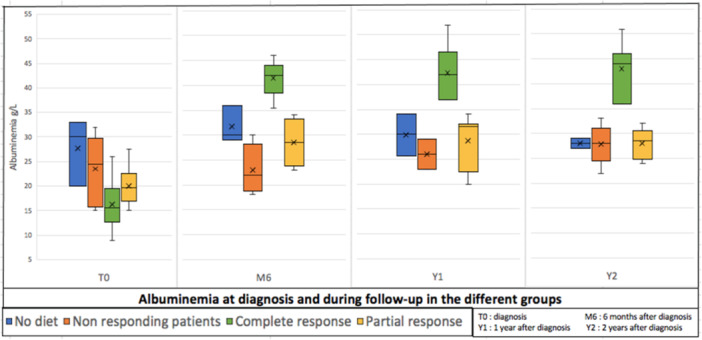
Albuminemia at diagnosis and during follow‐up in the different groups.

The median follow‐up duration for our cohort was 4.5 years (2–11). Three patients presented with mild symptomatic forms that did not require therapeutic intervention. For these three patients, symptoms spontaneously resolved with a median follow‐up of 2 years (1.5–4) even if albumin concentration remained under normal range (Figure 2). A total of 31 patients (91%) were prescribed a dietary management program, which consisted of a low LCT diet enriched with MCTs. Of these, 25 patients (81%) had a positive response. Among the six patients who did not respond to the diet (19%), three had poor compliance with clinical and biological recurrences (edema, hypoalbuminemia) sometimes necessitating albumin infusions. These three children had a median age at diagnosis of 8 years (7.9–12). One patient was a specific case of PIL associated with an IgE‐mediated cow's milk protein allergy. Anaphylactic shock under MCT‐enriched milk contraindicated its use and did not allow the strict implementation of the diet. An amino acid formula was finally chosen, but the child remained symptomatic. The two remaining patients presented with diffuse lymphatic involvement, either pulmonary or mediastinal, accompanied by lymphedema, and required second‐line drug treatments after failure of the dietary therapy. These two patients did not undergo genetic testing.

Among the 25 patients who responded to the dietary therapy, 10 patients (40%) achieved a complete response with normalization of albuminemia within a median period of 3.5 (0.6–4) months after starting the diet; albumin concentration remained sustained throughout all the follow‐up period. However, 15 patients (60%) had only a partial response with hypoalbuminemia but no need for further albumin infusions (Figure 1). Albuminemia at diagnosis and during follow‐up in the different groups is described in Figure 2. Among patients with a complete response (*n* = 10), a normal diet was successfully reintroduced in nine patients (90%) after a median duration of 1.8 years (1.3–2). No clinical or biological relapse was observed over a median follow‐up of 3.5 years (2.7–7.5). At the last consultation, the median serum albumin level was 45 g/L (38.5–46) in this group. These nine patients were therefore considered as cured, or at least in long‐term complete remission. The only good responder patient still on low LCT diet at the end of the study was a recent diagnosis. Among patients with a partial response (*n* = 15), normalization of the diet was achieved in five children (33%) after a median duration of 1 year (0.7–1.2). None of these children had clinical relapse, and albumin concentration remained stable between 28 and 34.5 g/L without any need for albumin infusion after a median follow‐up of 9 years (2.5–10) (Figure 1). At the end of the study, 50% of the cohort (*n* = 17) was receiving a normal diet.

The presence of associated lymphedema or a genetic variant was significantly more frequent in patients with a partial response compared with patients with complete response. Albuminemia was significantly lower at diagnosis in patients who had a complete response. Other clinical, biological or endoscopical characteristics were not significantly different between the two groups (Table 3).

**Table 3:** Comparison of diagnostic features in patients with complete response versus patients with partial response.

	Patients with complete response (*n* = 10)	Patients with partial response (*n* = 15)	
Age at diagnosis (months)	5 (4–7)	4.25 (1.5–25)	*p* = 0.062
Diagnostic delay (months)	1 (1–3)	1 (1–11.5)	*p* = 0.079
Edema	8 (80%)	13 (87%)	*p* = 0.68
Diarrhea	6 (60%)	6 (40%)	*p* = 0.33
**Lymphœdema**	**0 (0%)**	**6 (40%)**	***p*** = **0.022**
Ascite	2 (20%)	8 (53%)	*p* = 0.095
**Genetic variant identified**	**0 (0%)**	**5 (33%)**	***p*** = **0.041**
**Albuminemia**	**16.3 (13−19)**	**20.2 (17–24)**	***p*** = **0.004**
**Proteinemia**	**30 (25−32)**	**34.5 (31–41.5)**	***p*** = **0.009**
Lymphocytes	1.51 (0.6–2.3)	1.3 (1.1–6)	*p* = 0.23
IgG	0.61 (0.4–1.2)	1.3 (1.1–1.9)	*p* = 0.18
Suggestive endoscopic findings	5 (50%)	9 (60%)	*p* = 0.62
Lymphangiectasias on biopsies	5 (50%)	7 (47%)	*p* = 0.85

*Note*: All values are expressed as number (%) or median (interquartile range).The bold values indicate statistically significant results.

Abbreviation: IgG, immunoglobulin G.

Four patients required a second‐line treatment in our study (12% of the cohort): Octreotide (*n* = 1), Sirolimus (*n* = 2), and Calciparin then Octreotide (*n* = 1). One of these patients was initially classified as a nonresponder due to persistent significant clinical symptoms and the necessity of multiple albumin infusions 1 month after initiating the diet. Octreotide was introduced (increased up to 40 µg/kg/day) but was discontinued after 1 month due to its lack of efficacy. The evolution was finally favorable under dietary therapy alone with a complete response. Another patient with a partial response was treated with Sirolimus, aiming for the normalization of IgG levels to alleviate the child's challenging experience with weekly immunoglobulin infusions. While an initial improvement in laboratory parameters was observed (albuminemia at 34 g/L compared to 27 g/L before treatment and a near normalization of IgG), the loss of effectiveness prompted the discontinuation of treatment after 18 months. The other two patients presented with diffuse lymphatic involvement associated with lymphedema. After failure of the dietary treatment, second‐line treatment was started. For one patient administration of Calciparin for 1 year led to transient improvement for 5 years. Then because of a relapse Octreotide was introduced but discontinued after 1 year due to its ineffectiveness. The last patient received Sirolimus, discontinued after 6 months due to its inefficacy.

Anti‐infectious prophylaxis, either through monthly immunoglobulin infusions and/or three times a week oral Cotrimoxazole, was administered to 65% of the cohort (*n* = 23). In 59% of these cases (*n* = 13), prescription was justified by hypogammaglobulinemia alone, without any infectious context. In contrast, 35% of patients (*n* = 12) did not receive prophylaxis, and none of them presented severe infections during the follow‐up. None of the patients developed late complications such as lymphoma during the time of the study.

## 4 DISCUSSION

Thirty‐three out of the 36 French centers specialized in rare digestive diseases took part in the study (participation rate of 92%). Our population could therefore be considered representative of the French cohort of children with PIL followed between January 2010 and January 2022. To our knowledge, this is the largest cohort of children with PIL published to date. All the patients, except 3 who had very few symptoms, received a dietary treatment consisting of a strict low LCT diet, enriched with MCT and high protein intakes. Eighty‐one percent of them responded positively to this treatment and only 12% needed a second‐line drug therapy. During the follow‐up, a normal diet could be successfully reintroduced in 14 patients (45% of patients who received the diet), and nine of them were on long‐term clinical and biological remission at the end of the study. Associated lymphedema or an identified pathogenic genetic variant was present only in patients with a partial diet response; these two characteristics were associated with a chronic form of the disease.

The diagnosis of PIL remains challenging. In our study, the macroscopic appearance of gastroscopy and examination of biopsies supported the diagnosis in only 65% of cases, compared to 86% described in a cohort of 84 patients with PIL.^19^ When gastroscopy results are inconclusive for the diagnosis, we recommend performing either a colonoscopy to investigate ileal lymphangiectasia or a videocapsule which provides the advantage of delineating the extent of the damage^20^ to enhance diagnostic accuracy. In our cohort, these additional tests facilitated the diagnosis in five patients (14%) after a negative gastroscopy. A high long‐chain fatty acid meal the day before the endoscopy can also be proposed to enhance visualization of dilated lymphatic channels.^21^

First‐line treatment was a low LCT diet for all patients, except 3 (9%) who did not require any treatment because of a very mild and transient clinical form of the disease. The dietary response, which we defined as a clinical remission and no need for albumin infusion, was observed in 81% of patients in our study; a result comparable to the 78% success reported in the most recent cohort study by Prasad et al.^22^ This outcome underscores that the low LCT diet remains the cornerstone of treatment for PIL. Given the complexity of managing, dietary therapy must be accompanied by proper education, and we emphasize the importance of involving a dietician to educate families and ensure strict adherence to the prescribed diet.

Among good responders, 60% had a partial response, maintaining chronic hypoalbuminemia, while 40% achieved a complete response. In Kwon et al.'s study,^9^ a good dietary response was defined differently by the absence of symptoms and normalization of albumin levels, resulting in only one out of seven patients being classified as a good responder (14%); the remaining six patients (86%) received a second‐line treatment.

In our study, only four children (12% of the cohort) received second‐line treatments. Calciparin was the only intervention that led to a transient improvement in one patient, while sirolimus and octreotide were ineffective. When dietary therapy fails, several approaches have been described to achieve remission or clinical improvement, including surgical resection or embolization in cases of segmental disease,^9^, ^10^, ^11^, ^12^ as well as pharmacological treatments especially octreotide for diffuse intestinal lymphangiectasia and sirolimus for extensive disease (serous effusions).^8^, ^9^, ^19^, ^22^ The rationale for these therapies is based on their proposed mechanisms of action: octreotide reduces the absorption of triglycerides into the thoracic duct and reduces intestinal blood flow. On the other hand, sirolimus has antilymphangiogenic properties and is used with some efficacy in the treatment of vascular malformations with a lymphatic component.^23^ However, the literature reports inconstant clinical and biological improvements with both agents. Long‐term efficacy is difficult to assess due to the lack of randomized controlled trials in this rare disease and the limited follow‐up data available.^9^, ^22^ Currently, there are no predictive biomarkers of response described in the literature. Additionally, calcium channel blockers are not mentioned as a treatment option for PIL in existing studies.

The pathophysiology of PIL remains partially unknown. The current definition is based on the presence of peripheral lymphatic malformations associated in some cases with genetic defects affecting endothelial, connective tissue, immune, or metabolic functions.^24^ Nine patients (26% of the cohort) with a complete response tolerated the reintroduction of a normal diet well and were considered cured at the end of the study even if normalization of endoscopy and/or pathology was not documented. These patients appear to manifest a distinct form of the disease, characterized by less severity even if albuminemia concentration was lower at diagnosis, early and consistent normalization of albumin levels under diet and a better prognosis. None of them had associated lymphedema or an identified pathogenic genetic variant, two characteristics that were both associated with partial response to diet. Two patients who completely failed to respond to diet also had lymphedema and extensive disease; they had no genetic testing. These different profiles of response to diet could be related to completely different diseases. In cases of severe PIL with no or only poor response to dietary measures, especially in syndromic forms or in the presence of lymphedema, the exploration of genetic pathogenic variants may offer new insights into pathophysiology and second‐line therapeutic strategies.

Therapeutic recommendations regarding the necessary duration of the low LCT diet enriched with MCT have not been established yet. In most studies, diet normalization is not considered until biological signs of protein‐losing enteropathy persist, indicating on‐going lymphatic leakage. Nevertheless, in our study, five children with a partial response tolerated the gradual reintroduction of LCT after a median diet duration of 1 year. Albuminemia remained stable without the need for infusions, and the children exhibited normal growth. There is no specific albuminemia target threshold defined in the management of PIL. In our study the persistence of long‐term asymptomatic hypoalbuminemia was not associated with complications. At the end of the study, 50% of the cohort (*n* = 17) were on a normal diet. These findings suggest that attempting a gradual reintroduction of a normal diet in patients with favorable clinical evolution may be possible without waiting for albuminemia normalization. Nevertheless, this approach requires close clinical and biological monitoring during the reintroduction process and long‐term evolution needs to be studied.

Our study is constrained by the small size population and the retrospective nature of data collection, introducing several biases and instances of missing data. However, given the rarity of this pathology, conducting prospective studies is not feasible and our cohort of 34 patients is quite large in such a rare disease. Further studies are imperative to enhance the understanding of PIL.

## 5 CONCLUSION

This study represents the first description of a French national cohort of children with PIL, comprising the largest cohort published to date. As previously described, most patients (81% in our cohort) exhibited a good response to the dietary treatment. The pathophysiology of PIL remains elusive, and different evolutionary profiles seem to exist. Patients with a complete response to diet, 29% of the cohort, appeared to have a distinct form of the disease with a better prognosis. The presence of lymphedema and/or an identified genetic mutation were associated with a chronic condition with persistent hypoalbuminemia. Extension of genetic testing could offer new insights in pathophysiology and treatments. A normal diet may be successfully reintroduced in the absence of clinical symptoms, even if albuminemia remains subnormal.

## CONFLICT OF INTEREST STATEMENT

The authors declare no conflicts of interest.
